# Dot1l Aggravates Keratitis Induced by Herpes Simplex Virus Type 1 in Mice via p38 MAPK-Mediated Oxidative Stress

**DOI:** 10.1155/2021/6612689

**Published:** 2021-02-15

**Authors:** Shanshan Wan, Yiwen Zhou, Qiong Huang, Yanning Yang

**Affiliations:** Department of Ophthalmology, Renmin Hospital of Wuhan University, Wuhan, 430060 Hubei, China

## Abstract

**Background:**

Disruptor of telomeric silencing 1-like (Dot1l) plays a vital role in biological processes as a well-known methyltransferase. However, its role in herpes simplex virus type 1- (HSV-1-) infected keratitis remains unclear.

**Methods:**

*In vitro* and *in vivo* models were assessed to investigate the role of Dot1l in HSV-1 induced keratitis. C57BL/6 mice corneas were infected with HSV-1 for different days, with or without Dot1l inhibitor, to demonstrate the regulation of Dot1l in herpes simplex keratitis (HSK). Human corneal epithelial (HCE) cells were cultured and infected with HSV-1 to identify the molecular mechanisms involved.

**Results:**

In this study, we found that Dot1l was positively related to HSK. Inhibition of Dot1l with EPZ004777 (EPZ) alleviated corneal injury, including oxidative stress and inflammation *in vivo*. Similarly, the inhibition of Dot1l with either EPZ or small interfering RNA (siRNA) showed an inhibitory effect on HSV-1-induced oxidative stress and inflammation in HCE cells. Moreover, our study revealed that the expression of p38 MAPK was elevated after HSV-1 infection in HCE cells, and the inhibition of Dot1l could reduce the increased expression of p38 MAPK induced by HSV-1 infection *in vivo* and *in vitro*.

**Conclusion:**

Our results demonstrated that the inhibition of Dot1l alleviated corneal oxidative stress and inflammation by inhibiting ROS production through the p38 MAPK pathway in HSK. These findings indicated that Dot1l might be a valuable therapeutic target for HSK.

## 1. Introduction

Herpes simplex virus type 1 (HSV-1) is a highly prevalent virus [1] among the population. In humans, HSV-1 infection leads to encephalitis, paronychia, gingivitis, and blinding keratitis [2]. Eye disease caused by HSV-1 infection usually presents as herpes simplex keratitis (HSK), which accounts for 50–80% of ocular herpes [3]. HSK is threatening to ocular healthy, without adequate treatment, it may lead to progressive corneal opacity and poor eyesight [4]. Previous studies have shown that HSK is the leading cause of infectious blindness in the developed world [2]. However, the effect treatment for HSK is still limited.

The overproduction of reactive oxygen species (ROS) leads to oxidative stress, which is recognized as one of the most important factors in the pathogenesis of corneal diseases [5]. The generation of ROS is usually through the mitochondrial electron transport chain under physiological conditions, and it plays a key role in activating cellular factors or signaling for survival. However, the overproduction of ROS may cause oxidative damage to the cell, including lipid oxidative damage to DNA, intracellular oxidative modification of proteins, and peroxidation of the membrane [6, 7]. Up to now, although oxidative stress was widely participated in ocular diseases, its role in the progression of HSK was still unknown. In this study, we focused on the effect of oxidative stress in the pathogenesis of HSK and its possible mechanism.

Disruptor of telomeric silencing-1 like (Dot1l) protein specifically catalyzes the methylation of histone H3 on Lys79 (H3K79) in targeted gene and is found to be related with oxidative stress. Besides, it has been reported that Dot1l is related to many biological processes, such as DNA damage response, cell cycle progression, somatic reprogramming, transcriptional regulation, and embryonic cell development [8, 9]. As a conservative protein, Dot1l is widely expressed in different species [10]. However, the role of Dot1l in HSK remains unclear. In the present study, we detected the role of Dot1l in HSK. We also investigated the potential mechanisms involved in the Dot1l-mediated generation of ROS.

## 2. Material and Methods

### 2.1. Animal

All C57BL/6 mice (male; weight, 60-80 g; age, 6-8weeks) were provided by the Center of Experimental Animals in the Medical College, Wuhan University. This project was approved by the committee of experimental animals of Wuhan University, and the procedures were carried out in accordance with routine animal-care guidelines. All procedures complied with the Guidelines for the Care and Use of Laboratory Animals. Before surgery procedures, mice were anesthetized intraperitoneally with sodium pentobarbital (50 mg/kg) and then placed on a homeothermic table to maintain core body temperature at 37°C.

### 2.2. Virus

The virus HSV-1 KOS strain had a titer of 2 × 10^7^ pfu/ml before use, based on a previous study [11, 12]. The virus was produced by Vero cells. Mice were anesthetized intraperitoneally and scratched on the mouse corneal epithelium with the back of the blade of the No. 5 surgical blade. Subsequently, 5 *μ*l of a solution containing HSV-1 (KOS strain; 10^5^ spot forming units (pfu)) was spotted and retained for 10 s on the cornea, and the eyelids were closed and massaged for 30 s to allow the virus fluid to sufficiently contact the cornea. After surgery, 0.5% gentamicin eye drops were used to avoid bacterial infection.

### 2.3. Experimental Design and Groups

Mice were infected with HSV-1 to establish a corneal HSV-1 infection model. Then, they were sacrificed prior to corneal infection or at 1, 3, and 7 days postinfection (dpi). The inoculated eyes (five mice in each group) were enucleated and immediately frozen in liquid nitrogen or 4% formalin fixative for the following experiments.

All mice were divided into different groups (*n* = 5): control group, different infection time of HSV-1 group (1, 3, and 7days), Dot1l inhibitor group (10 mg/kg, 50 mg/kg), and dimethyl sulfoxide (DMSO) group. In the HSV-1 group, only the right eyes were scratched and then infected with HSV-1 on different days. In the Dot1l inhibitor group, after the right eyes were scratched and infected, the mice were administered with EPZ004777 by dissolving in DMSO via subconjunctival injection once daily. In the DMSO group, after infected with HSV-1, the mice were administered with equal DMSO as the control. The concentration of DMSO in the Dot1l inhibitor group and DMSO group was 0.1%. The score of corneal opacity was based on the opaque area of the cornea [13]: 0, the corneal stroma was clear and transparent; 1, mild corneal haze; 2, moderate corneal opacity with iris visible; 3, severe corneal opacity with indistinct distinguish the position of pupil; and 4, severe corneal opacity with invisible intraocular structure.

### 2.4. Cell Culture and Treatment

Human corneal epithelial (HCE) cells (provided by Shandong Eye Institute, Qingdao, Shandong, China) were cultured in DMEM with 10% fetal bovine serum (Gibco), 1% penicillin G (Gibco), and streptomycin sulfate (Solarbio) at 37°C, and 5% CO2. About 80% confluence, the cells were replaced in serum-free DMEM for 24 hours and treated with HSV-1 for 3, 6, and 12 hours.

### 2.5. Small Interfering RNA (siRNA) Transfection

HCE cells were transfected with either small interfering RNA against the targeting gene or with nontargeting siRNAs (Santa Cruz, CA, USA) at a concentration of 100 nM, and nontargeting siRNAs served as a negative control (NC) for 48 h using Lipofectamine 3000 reagent. The effects of siRNA were assessed using western blot or RT-PCR.

### 2.6. Histological Examinations

After the tissues were fixed in 4% paraformaldehyde, they were embedded in paraffin and incised with an average thickness of 4 *μ*m. Then, the sections were deparaffinized, hydrated, and stained with hematoxylin and eosin (H&E) in order to assess histopathological corneal injury. Morphological assessments were observed by two experienced pathologists who were unaware of the experimental design.

### 2.7. Immunofluorescence Staining

After the tissues were fixed in 4% paraformaldehyde, they were embedded in paraffin and incised with an average thickness of 4 *μ*m. For immunofluorescence staining, the sections were incubated with diluted CD31 primary antibody (BD, New Jersey, USA) overnight at 4°C. After washing with PBS, fluorescence-conjugated secondary antibody was added and incubated at 37°C for 2 h. Then, DAPI (Invitrogen, United Kingdom) was added for 5 min to visualize the nuclei. Finally, the signal was observed under the fluorescence microscope (Olympus, Japan).

### 2.8. RT-PCR

RNAiso Plus (TaKaRa Biotech, Dalian, China) was used to extract total RNA from frozen corneal tissues according to the instructions provided by the manufacturer. Subsequently, the PrimeScript™ RT Reagent Kit (TaKaRa Biotech) was used for reverse transcription into cDNA. In all PCR experiments, the expression of GAPDH was used as the internal reference. The qRT-PCR analysis was performed using the ABI ViiA7DX System (Foster City, CA, USA). The qRT-PCR primers for the specific target genes (listed below) were designed and synthesized by TaKaRa Biotech. Routine qRT-PCR for the following genes and GAPDH was performed as follows: 94°C for 3 min, followed by 30 cycles (25 cycles for GAPDH) at 94°C for 30 s, 55°C for 30 s, and 72°C for 1 min. The primers included as follows.

M-IL-1*β*: 5′-CGCAGCAGCACATCAACAAGAGC-3′ (F)

5′-TGTCCTCATCCTGGAAGGTCCACG-3′ (R)

M-MMP1: 5′-TCCACAGTTGACAGGCTCCG-3′ (F)

5′-GGCACTCCACATCTTGGTTTTC-3′ (R)

M-MMP2: 5′-GACCCTGAAACCGTGGATGAT-3′ (F)

5′-GCCATCAGCGTTCCCATACTT-3′ (R)

M-IL-6: 5′-TGATGGATGCTACCAAACTGGA-3′ (F)

5′-TGTGACTCCAGCTTATCTCTTGG-3′ (R)

M-MMP9: 5′-AGTTTGGTGTCGCGGAGCA-3′ (F)

5′-AATGGGCATCTCCCTGAACG-3′ (R)

M-GAPDH: 5′-ATGGGTGTGAACCACGAGA-3′ (F)

5′-CAGGGATGATGTTCTGGGCA-3′ (R)

H-IL-1*β*: 5′-GCTGATGGCCCTAAACAGATGAA-3′ (F)

5′-TCCATGGCCACAACAACTGAC-3′ (R)

H-MMP1: 5′-GGACCATGCCATTGAGAAAGC-3′ (F)

5′-TTGTCCCGATGATCTCCCCT-3′ (R)

H-MMP2: 5′-TCAATGGCAAGGAGTACAACAGC-3′ (F)

5′-CACCTTCTGAGTTCCCACCAA-3′ (R)

H-IL-6: 5′-AAGCCAGAGCTGTGCAGATGAGTA-3′ (F)

5′-TGTCCTGCAGCCACTGGTTC-3′ (R)

H-MMP9: 5′-TCGACGTGAAGGCGCAGAT-3′ (F)

5′-AGAAGCGGTCCTGGCAGAAATA-3′ (R)

H-GAPDH: 5′-TCAAGAAGGTGGTGAAGCAGG-3′ (F)

5′-TCAAAGGTGGAGGAGTGGGT-3′ (R)

### 2.9. Western Blotting

Proteins from cornea tissue were extracted and quantified using the bicinchoninic acid method. Then, equal concentrations of protein were separated on 10% sodium dodecyl sulfate polyacrylamide gel electrophoresis gels and then transferred to a nitrocellulose membrane. Primary antibodies against Dot1l (ab64077), catalase (ab209211), SOD1 (ab51254), SOD2 (ab68155), p38 (ab31828), p-p38 (ab178867), and *β*-actin (ab8226) were purchased from Abcam (dilution 1 : 1000). *β*-Actin was used as a loading control to ensure equal loading. Subsequently, membranes were washed twice with PBS and then incubated with goat anti-rabbit or goat anti-mouse horseradish peroxidase-conjugated immunoglobulin G secondary antibody (1 : 2,000; ZDR 5306, ZDR 5307, ZSGB BIO, Beijing, China) at room temperature for 1 h. Specific bands were then visualized using Immobilon Western Chemiluminescence HRP substrate (Merck Millipore, Darmstadt, Germany). Optical densities were detected using Quantity One software (Bio-Rad, Hercules, CA, USA).

### 2.10. Measurement of MDA and SOD

The detection of SOD activity and MDA content was performed through the commercial kits (Nanjing Jiancheng Bioengineering Institute, China). Cell lysates from *in vivo* and *in vitro* experiments were used to examine the level of MDA and SOD activity. The specific procedures were according with the manufactory's direction.

### 2.11. ROS Production Detection

Intracellular ROS levels were determined using the Reactive Oxygen Species Assay Kit (Nanjing Jiancheng Bioengineering Institute, Nanjing, China) based on our previous study [14]. Briefly, cells pretreated with different reagents were incubated with 20 *μ*M dichloro-dihydro-fluorescein diacetate (DCFH-DA) in Hanks' balanced salt buffer for 30 min at 37°C. The ROS level was quantified using flow cytometry.

### 2.12. Detection for the Production of H_2_O_2_

H_2_O_2_ production was measured by Amplex Red in HCE cells and corneal tissues, based on our previous study [14]. To detect the corneal tissue H_2_O_2_ levels, the corneas were first perfused with HEPES-modified Tyrode's solution and homogenized. The hydrogen peroxide in the homogenate was measured using Amplex Red (100 *μ*M, Invitrogen) with 10 U/mL horseradish peroxidase. Fluorescent readings were obtained from the mice cornea after 1 h of incubation at 37°C, and the values were normalized to the protein amount as measured by a Bradford assay. The Amplex Red reagent is a colorless substrate that reacts with H_2_O_2_ with a 1 : 1 stoichiometry to produce the highly fluorescent resorufin (excitation/emission maxima = 570/585 nm).

### 2.13. Statistical Analysis

Data are presented as mean ± standard error of the mean (SEM). The means of the different groups were compared using one-way analysis of variance (ANOVA) and the Student–Newman–Keuls test. Differences were considered to be statistically significant when *P* < 0.05.

## 3. Results

### 3.1. Clinical Course of Keratitis and Oxidative Stress after Corneal HSV-1 Infection

**Figure 1:**
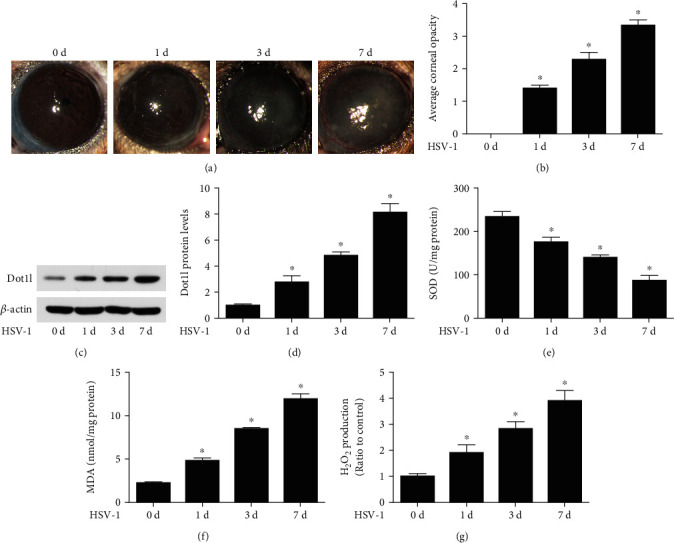
Dot1l was elevated in HSK progression. The corneal images were taken using a slit lamp (a) and calculated for opacity score (b) after HSV-1 infection 1, 3, and 7 days. The levels of Dot1l protein (c) were detected after HSV-1 infection 1, 3, and 7 days, and quantification of Dot1l expression (d) was determined in fold change relative to the 0 d group. SOD activity (e), MDA content (f), and H2O2 production (g) were also detected after HSV-1 infection 1, 3, and 7 days. Data were expressed as means ± SD (*n* = 5). ^∗^*P* < 0.05 versus 0 d group. Experiments were repeated 3 times.

Corneal morphology images indicated that C57BL/6 mice were susceptible to HSV-1 infection and developed typical keratitis at 7 dpi, which lead to the most serious corneal opacity (Figures 1(a) and 1(b)). To explore the role of Dot1l in HSK, its expression was measured at 0, 1, 3, and 7 dpi in mice. Compared with 0 day, HSV-1-infected corneas displayed obviously elevated Dot1l expression at 1, 3, and 7 dpi, with the highest expression at 7 dpi (Figures 1(c) and 1(d)). Also, we found that oxidative stress was related to the HSK. The results suggested that SOD activity (Figure 1(e)) continued to decrease and MDA content (Figure 1(f)), and H2O2 production (Figure 1(g)) continued to increase in the course of HSK progressed. Overall, these results indicated that Dot1l expression and oxidative stress were related to the progression of HSK.

### 3.2. Dot1l Inhibition Alleviated HSV-1-Induced Keratitis

**Figure 2:**
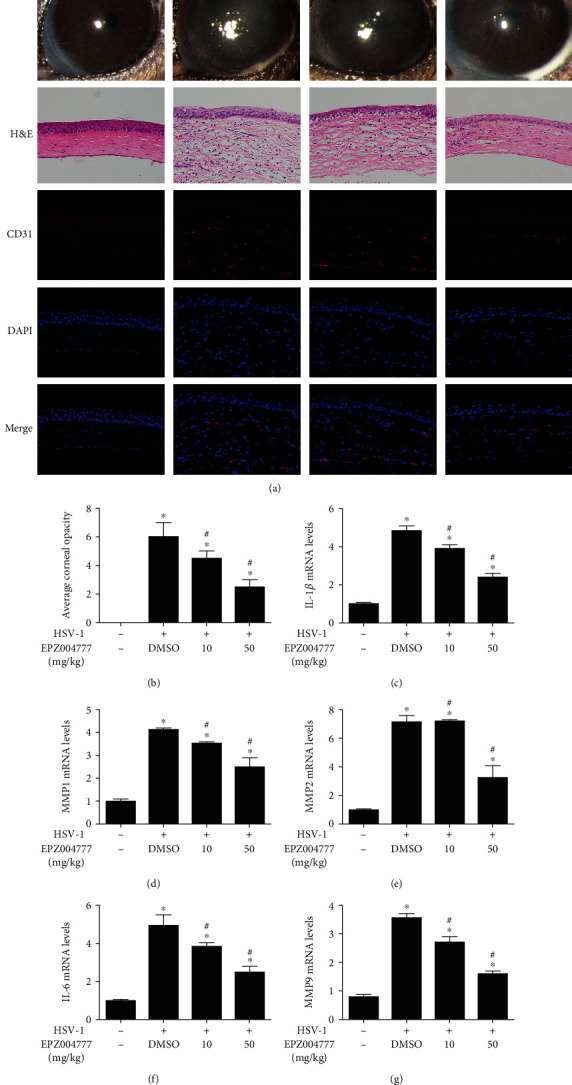
Dot1l inhibition prevented keratitis induced by HSV-1 infection. The corneal images were taken using a slit lamp (a) and calculated for opacity score (b) at HSV-1 7dpi in mice, with or without Dot1l inhibitor EPZ004777 (10 and 50 mg/kg) treatment. H&E staining (×400) and immunofluorescence staining (×400) for CD31 were also performed (a). The mRNA levels of proinflammatory factors (c–g), including IL-1*β*, MMP-1, MMP-2, IL-6, and MMP-9, were detected at HSV-1 7 dpi in mice, with or without Dot1l inhibitor EPZ004777 (10 and 50 mg/kg) treatment. Data were expressed as means ± SD (*n* = 5). ^∗^*P* < 0.05 versus control group; ^#^*P* < 0.05 versus DMSO group. Experiments were repeated 3 times.

Treatment with different concentrations (10 and 50 mg/kg) of Dot1l inhibitor via subconjunctival injection could alleviate corneal injury and opacity (Figures 2(a) and 2(b)) induced by HSK at 7 dpi. H&E staining showed that the HSV-1-infected cornea had extensive pathologic vessel growth at 7 dpi (Figure 2(a)), however, Dot1l inhibition via subconjunctival injection obviously decreased neonatal corneal vessels. CD31 immunofluorescent staining results showed that the inhibition of Dot1l could reduce the elevated CD31 expression induced by HSV-1 keratitis (Figure 2(a)). The mRNA levels of proinflammatory factors, including IL-1*β*, MMP-1, MMP-2, IL-6, and MMP-9, were increased at 7 dpi (Figures 2(c)–2(g)), however, Dot1l inhibition could reduce mRNA levels of these proinflammatory factors. These results suggested that Dot1l inhibition alleviated HSK.

### 3.3. Dot1l Inhibition Attenuated Oxidative Stress Induced by HSV-1 Keratitis

**Figure 3:**
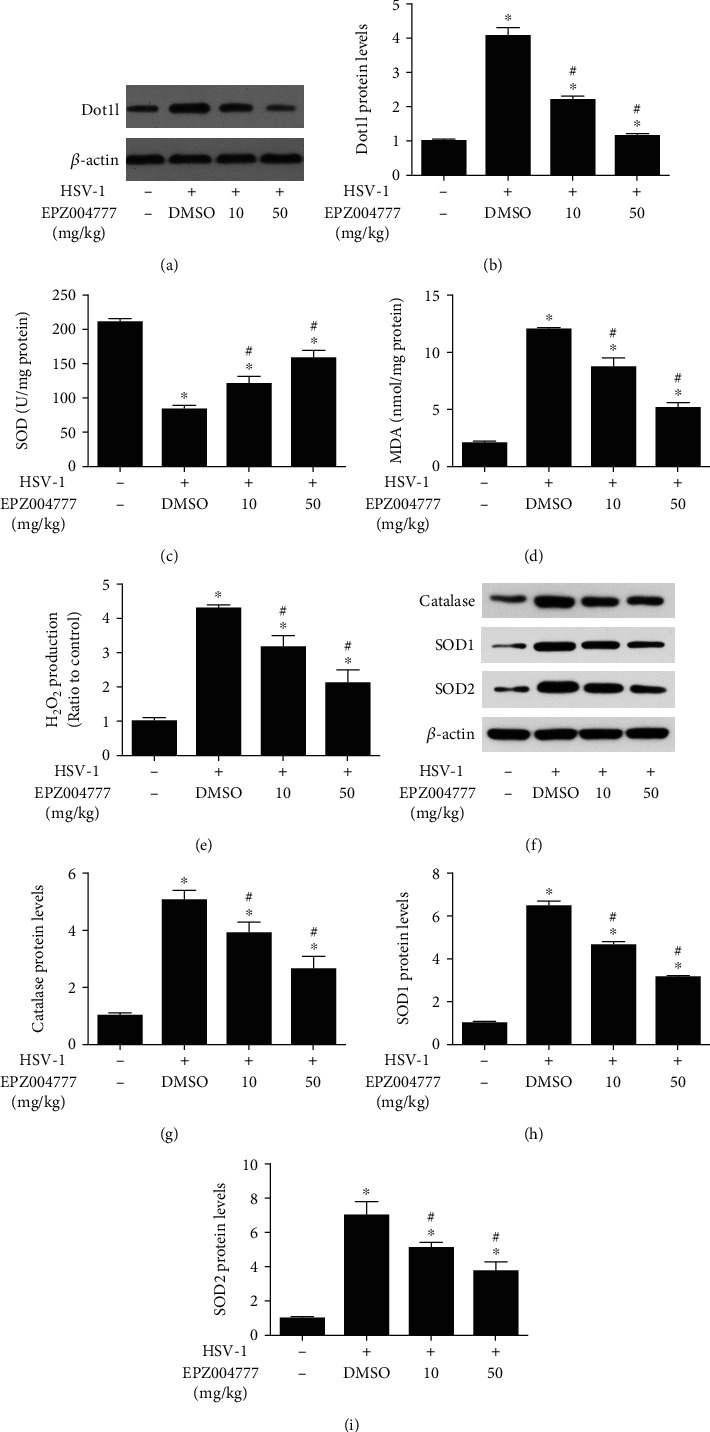
Dot1l inhibition prevented oxidative stress induced by HSV-1 infection. Western blot was performed for the expression of Dot1l (a) after HSV-1 infection 7 days, with or without Dot1l inhibitor EPZ004777, and quantification of their expression (b) in fold change relative to the control group. SOD activity (c), MDA content (d), and H2O2 production (e) were also detected at HSV-1 7dpi in mice, with or without Dot1l inhibitor EPZ004777. The expression of catalase, SOD1, and SOD2 were also detected by western blot and quantification of their expression (f–i) in fold change relative to the control group. Data were expressed as means ± SD (*n* = 5). ^∗^*P* < 0.05 versus control group; ^#^*P* < 0.05 versus DMSO group. Experiments were repeated 3 times.

WB results showed that HSV-1 keratitis could stimulate Dot1l expression, which were inhibited by EPZ004777 at different concentration (Figures 3(a) and 3(b)). Next, we investigated the relationship of Dot1l and oxidative stress induced by HSV-1 keratitis. It was indicated that the decreased SOD level (Figure 3(c)) and the increased MDA content (Figure 3(d)), H_2_O_2_ production (Figure 3(e)) and induced by HSV-1 keratitis could be reversed by Dot1l inhibitor. WB results also indicated that catalase, SOD1, and SOD2 expression were increased after HSV-1 infected keratitis, and the inhibition of Dot1l could decrease their expression (Figures 3(f)–3(i)). Overall, these results indicated that Dot1l inhibition might reduce oxidative stress induced by HSV-1 keratitis.

### 3.4. Oxidative Stress Induced by HSV-1 Infection Depends on Dot1l in HCE Cells

**Figure 4:**
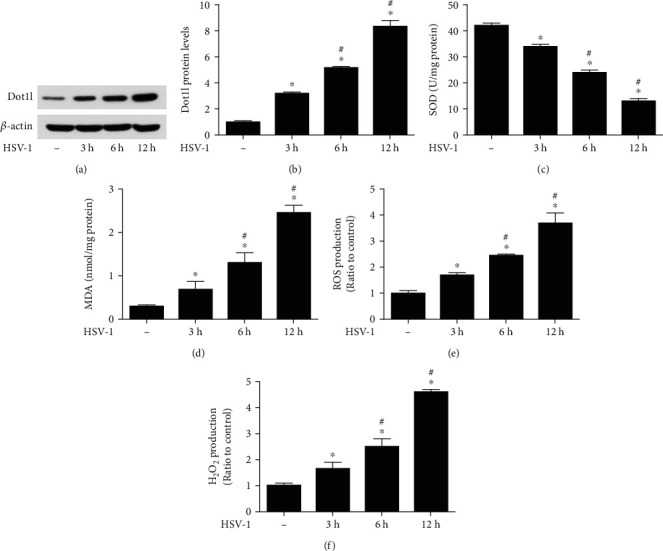
Dot1l expression was elevated induced by HSV-1 infection 3, 6, and 12 hours in HCE cells. Western blot was performed for the expression of Dot1l (a) after HSV-1 infection 3, 6, and 12 hours in HCE cells, and quantification of their expression (b) in fold change relative to the control group. SOD activity (c), MDA content (d), ROS (e), and H2O2 production (f) were also detected at HSV-1 infection 3, 6, and 12 hours in HCE cells. Data were expressed as means ± SD (*n* = 5). ^∗^*P* < 0.05 versus control group; ^#^*P* < 0.05 versus HSV-1 3 h group. Experiments were repeated 3 times.

First, we determined whether different HSV-1 infection time affected Dot1l expression in HCE cells. Its expression in HSV-1 infection groups, 3, 6, and 12 h, was significantly elevated compared with that observed in the control group, with the more obvious effects at 12 h post-HSV-1 infection (Figures 4(a) and 4(b)). We also found that SOD activity (Figure 4(c)) continued to decrease in the course of HSV-1-infected HCE cells. However, MDA content (Figure 4(d)), ROS (Figure 4(e)), and H_2_O_2_ production (Figure 4(f)) were significantly increased in HCE cells in response to HSV-1 infection.

### 3.5. Inhibition of Dot1l Decreased Oxidative Stress Induced by HSV-1 in HCE Cells

**Figure 5:**
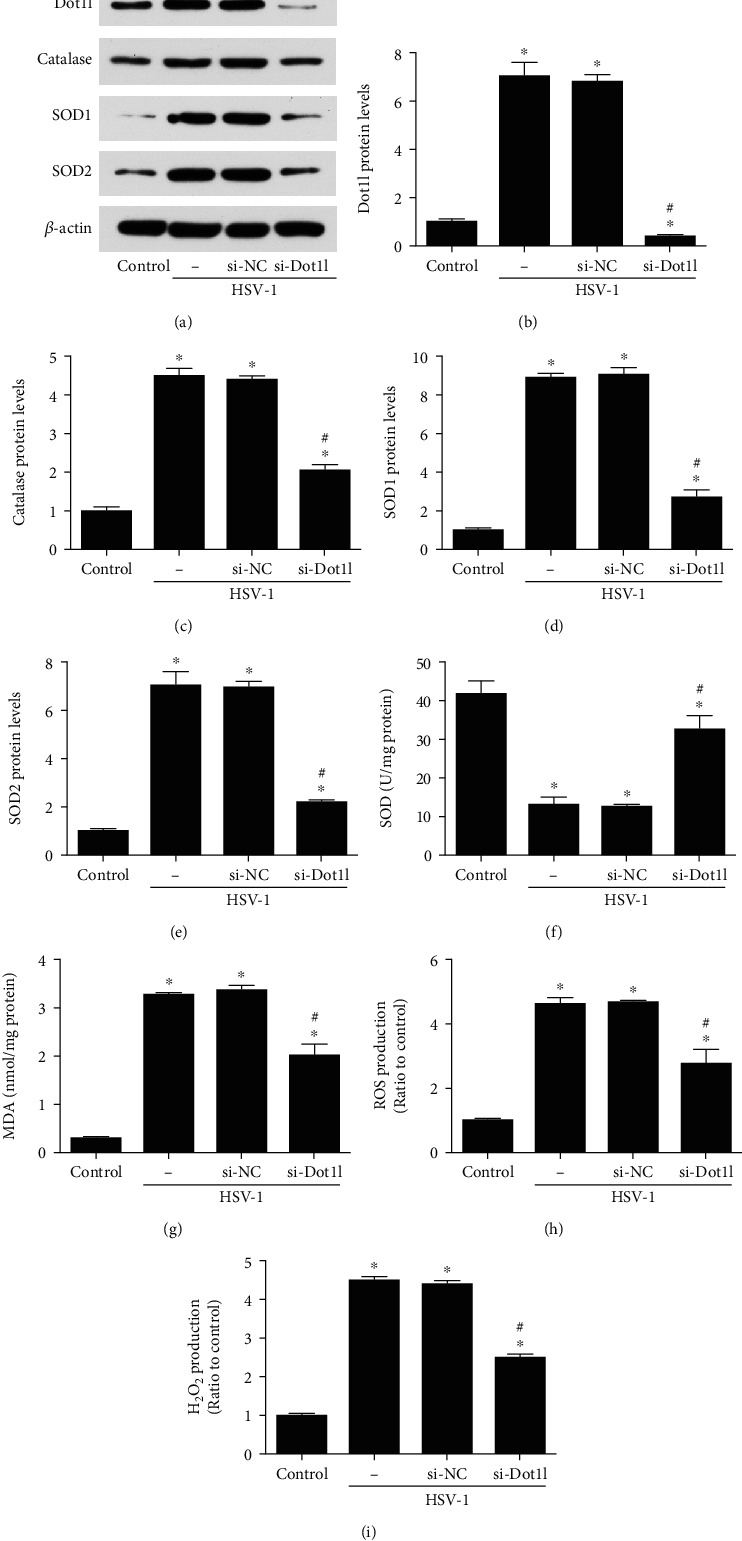
Inhibition of Dot1l alleviated oxidative stress induced by HSV-1 infection 12 hours in HCE cells. The expression of Dot1l, catalase, SOD1, and SOD2 were evaluated through western blot after transfection with siRNA against Dot1l (a–e). SOD activity (f), MDA content (g), ROS (h), and H2O2 production (i) were also detected at HSV-1 infection 12 hours in HCE cells, with siRNA against Dot1l. Data were expressed as means ± SD (*n* = 5). ^∗^*P* < 0.05 versus control; ^#^*P* < 0.05 versus si-NC. Experiments were repeated 3 times.

WB results showed that catalase, SOD1, and SOD2 expression were increased after HSV-1 infection, and the siRNA against Dot1l could decrease their expression in HCE cells (Figures 5(a)–5(e)). Also, it was found that the decreased SOD level (Figure 5(f)) and the increased MDA content (Figure 5(g)), ROS (Figure 5(h)), and H_2_O_2_ production (Figure 5(i)) induced by HSV-1 infection could be reversed by si-Dot1l in HCE cells.

### 3.6. Dot1l Regulated the Activation of p38 MAPK in HSV-1-Infected HCE Cells

**Figure 6:**
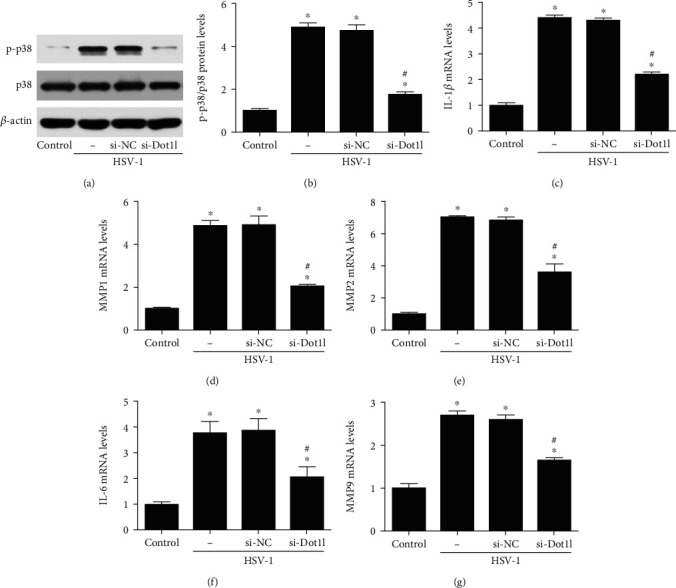
Inhibition of Dot1l alleviated p38 MAPK activation and inflammation induced by HSV-1 infection 12 hours in HCE cells. The activation of p38 MAPK was evaluated through western blot after transfection with siRNA against Dot1l (a–d). The mRNA levels of proinflammatory factors, including IL-1*β*, MMP-1, MMP-2, IL-6, and MMP-9 (c–g), were also detected at HSV-1 infection 12 hours in HCE cells, with siRNA against Dot1l. Data were expressed as means ± SD (*n* = 5). ^∗^*P* < 0.05 versus control; ^#^*P* < 0.05 versus si-NC. Experiments were repeated 3 times.

It was reported that p38 MAPK was activated in experimental keratitis *in vivo* and *in vitro*. In this study, we found that phosphorylated p38 (p-p38) was obviously elevated after HSV-1 infection (Figures 6(a) and 4(b)); however, the total p38 level was not changed in different groups. Interestingly, the increased p-p38 level was largely suppressed by knockdown of Dot1l compared with the si-NC group. In addition, the mRNA levels of proinflammatory factors, including IL-1*β*, MMP-1, MMP-2, IL-6, and MMP-9 (Figures 6(c)–6(g)), were elevated after HSV-1 infection in HCE cells, and si-Dot1l could alleviate their mRNA levels. These results indicated that p38 MAPK was activated by HSV-1 infection, and the inhibition of Dot1l could suppress p38 MAPK activation in HCE cells.

### 3.7. p38 MAPK Inhibitor Reduced Oxidative Stress in HSV-1-Infected HCE Cells

**Figure 7:**
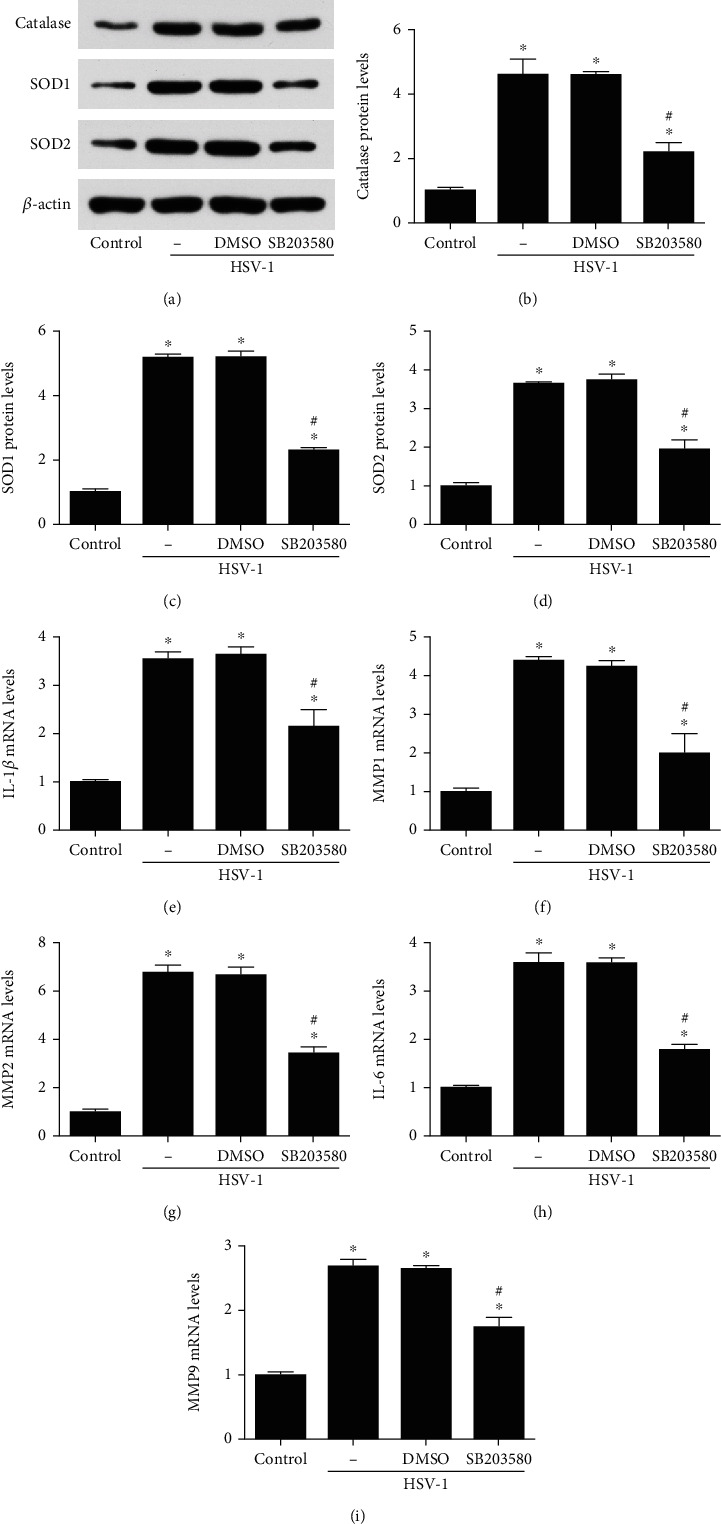
Inhibition of p38 MAPK expression alleviated oxidative stress and inflammation induced by HSV-1 infection 12 hours in HCE cells. The expression of catalase, SOD1, and SOD2 (a–d) were evaluated through western blot after pretreatment with SB203580 (1 *μ*M) for 1 hour in HCE cells before HSV-1 infection. The mRNA levels of proinflammatory factors, including IL-1*β*, MMP-1, MMP-2, IL-6, and MMP-9 (e–i), were also detected after treatment with SB203580 (1 *μ*M) for 1 hour in HCE cells before HSV-1 infection. Data were expressed as means ± SD (*n* = 5). ^∗^*P* < 0.05 versus control; ^#^*P* < 0.05 versus DMSO. Experiments were repeated 3 times.

WB results showed that after HSV-1 infection in HCE cells, the increased catalase, SOD1, and SOD2 expression were reversed by p38 MAPK inhibitor (Figures 7(a)–7(d)). In addition, the mRNA levels of proinflammatory factors, including IL-1*β*, MMP-1, MMP-2, IL-6, and MMP-9(Figures 7(e)–7(i)), were reduced in response to the inhibition of p38 MAPK. These results suggested that p38 MAPK could alleviate oxidative stress induced by HSV-1 infection in HCE cells.

### 3.8. Inhibition of Dot1l Attenuated p38 MAPK Activation Induced by HSV-1 Keratitis

**Figure 8:**
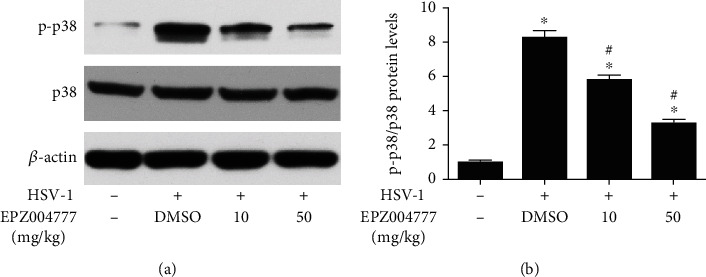
EPZ004777 alleviated p38 MAPK activation induced by HSV-1. Western blot was performed for the activation of p38 MAPK (a) after treatment with EPZ004777 (10 and 50 mg/kg) at HSV-1 infection 7 days, and quantification of their expression (b) in fold change relative to the control group. Data were expressed as means ± SD (*n* = 5). ^∗^*P* < 0.05 versus control; ^#^*P* < 0.05 versus DMSO. Experiments were repeated 3 times.

The effects of Dot1l inhibition on p38 MAPK activation observed *in vitro* also needed to be verified *in vivo*. As shown, p-p38 MAPK was increased at 7 dpi, however, their expression was inhibited by treatment with Dot1l inhibitor (Figures 8(a) and 8(b)).

## 4. Discussion

In the present study, we focused on the effect of Dot1l in the HSK model and investigated the underlying mechanism. We found that Dot1l played an important role in HSK in mice. The results showed that the inhibition of Dot1l could alleviate corneal injury induced by HSV-1 infection in mice. Besides, oxidative stress induced by HSV-1 infection relied on Dot1l in HCE cells, and inhibition of Dot1l using siRNA blocked the inflammation and oxidative stress induced by HSV-1 infection in HCE cells. Furthermore, we also found that ROS generation was modulated by Dot1l through p38 MAPK activation. Therefore, our findings demonstrated that Dot1l might be a therapeutic target for HSK, while EPZ004777 might be an effective therapeutic agent for corneal injury induced by HSV-1 infection.

Corneal lesions caused by HSV-1 involve the direct effect of the virus and the immunoinflammatory response triggered by virus particles [15]. HSV-1 infection model has been studied in different animals to understand the pathogenesis, biology, and immune response, and the pattern of infection was different due to the animal species, age, and genotype and viral serotype and strain [16]. In this study, we observed that C57BL/6 mice were susceptible to HSV-1 infection and developed typical human stromal keratitis at 7 dpi, which lead to the most serious corneal opacity. Next, we chose 7 days as the observation time point in the following experiments. Morphological results of H&E staining showed that the HSV-1 infected cornea had extensive pathologic vessel growth and CD31 positive cells at 7 dpi. These results were consisted with the previous study, suggesting that HSV-1 infection induced corneal keratitis in mice [17].

Dot1l, as the histone methyltransferase, is correlated with mammalian development. A previous study showed that Dot1l was overexpressed in prostate cancer and associated with poor outcome. Chemical or genetic inhibition of Dot1l impaired the viability of androgen receptor-positive prostate cancer cells [18]. Other studies found that Dot1l epigenetically promoted the transcription of c-Myc via H3K79me2, while silence or inhibition of Dot1l induced cell cycle arrest in colorectal cancer cells, suggesting that Dot1l inhibitor might be a potential drug for the treatment of colorectal cancer [19]. Until now, Dot1l has been widely studied for cancer pathogenesis and development, however, the role and function of Dot1l in HSK still remain unknown. In this study, it was indicated that Dot1l inhibition could reduce elevated neonatal vessel growth, CD31 expression, and mRNA levels of these proinflammatory factors induced by HSV-1 infection, which suggested the vital role of Dot1l on the regulation of HSK progression.

Although oxidative stress plays a key role in the regulation of many biological processes, including intracellular signaling [20], it can also induce serious cellular damage under adverse condition. The imbalance between free-radical-generating and radical-scavenging systems could lead to oxidative stress, which is associated with noninfective diseases and infective diseases. A previous study has been demonstrated that ROS-induced oxidative injury is involved in the pathogenesis of fungal keratitis in mice [21]. However, whether oxidative stress participated in the HSK was still unknown. In this study, the results showed that the inhibition of Dot1l could decrease the elevated Catalase, SOD1, and SOD2 expression induced by HSV-1, as well as MDA content, H_2_O_2_ production, and the proinflammatory cytokines. In response to si-Dot1l in HCE cells, it was found that the decreased SOD level and the increased MDA content, ROS, and H_2_O_2_ production induced by HSV-1 could be reversed in HSV-1 infected HCE cells. These results indicated that the inhibition of Dot1l could alleviate HSK through the regulation of oxidative stress.

Oxidative stress might also activate MAPK signaling pathways, as elevated ROS could selectively activate ERKs, JNKs, or p38 MAPKs [22]. A previous study has shown that blockage of p38 MAPK activity may decrease ROS-mediated injury in the pathogenesis of fungal keratitis [21]. However, whether p38 MAPK participated in the HSK was still unknown. In this study, we found that p38 MAPK was activated by HSV-1induced infection, and inhibition of Dot1l could suppress p38 MAPK activation in HCE cells. Besides, with the p38 MAPK inhibitor, we further demonstrated that the expression of catalase, SOD1, SOD2, and the mRNA levels of proinflammatory factors, including IL-1*β*, MMP-1, MMP-2, IL-6, and MMP-9, was reduced in response to the inhibition of p38 MAPK activation. In addition, the results also showed that the activation of p38 MAPK was inhibited by treatment with Dot1l inhibitor in mice cornea infected by HSV-1.

## 5. Conclusion

In summary, we revealed that the inhibition of Dot1l protected the cornea against HSK and prevented corneal injury through modulating p38 MAPK-mediated ROS production. Overall, these results indicated that Dot1l might be a novel therapeutic target for HSK.

## Data Availability

The datasets used and analyzed during the current study are available from the corresponding author on reasonable request.

## Abbreviations

Dot1l: Disruptor of telomeric silencing 1-like
HSV-1: Herpes simplex virus type 1
HSK: Herpes simplex keratitis
HCE: Human corneal epithelial
EPZ: EPZ004777
siRNA: Small interfering RNA
ROS: Reactive oxygen species
H3K79: Histone H3 on Lys79
PFU: Plaque-forming units
Dpi: Postinfection
DMSO: Dimethyl sulfoxide
NC: Negative control
H&E: Hematoxylin and eosin
H_2_O_2_: Hydrogen peroxide
SOD: Superoxide dismutase
MDA: Malondialdehyde
DMEM: Dulbecco's modified Eagle's medium
FBS: Foetal bovine serum
CO2: Carbon dioxide
WB: Western blot
qRT-PCR: Real-time quantitative reverse transcription-polymerase chain reaction
MMP: Matrix metalloproteinase
MAPK: Mitogen-activated protein kinases.
